# Comparative Evaluation of Efficacy and Safety of the Diode Laser (980 nm) and Sclerotherapy for the Treatment of Oral Pyogenic Granuloma

**DOI:** 10.1155/2022/8269221

**Published:** 2022-09-17

**Authors:** Peeyush Shivhare, Naqoosh Haidry, Neha Sah, Ajay Kumar, Abhishek Gupta, Ankur singh, Mohan Raju Penumatcha, Shalini Subramanyam

**Affiliations:** ^1^Department of Dentistry, All India Institute of Medical Sciences, Patna 801507, India; ^2^Department of Oral and Maxillofacial Surgery, Dental College Azamgarh, Azamgarh 276128, India; ^3^Department of Oral Medicine and Radiology, Faculty of Dental Sciences, Institute of Medical Sciences, Banaras Hindu University, Varanasi 221005, India; ^4^Department of Oral Medicine and Radiology, Chitwan Medical College, Bharatpur, Chitwan 44207, Nepal; ^5^Department of Oral Medicine and Radiology, Narsinhbhai Patel Dental College and Hospital, Visnagar 384315, India; ^6^Private Dental Clinic, Secunderabad 500003, India; ^7^Private Dental Clinic, Bangalore 560038, India

## Abstract

**Background:**

Pyogenic granuloma (PG) is a tumor-like, non-neoplastic lesion of the soft tissue that commonly appears in the oral cavity. Various treatment modalities have been discussed, including surgical excision, cryosurgery, curettage, electrodessication, corticosteroid injection, sclerotherapy, and lasers. This observational retrospective study compared effectiveness between diode lasers and sclerotherapy for PG treatment.

**Materials and Methods:**

From July 2016 to January 2021, data of oral PG cases treated with sclerotherapy and diode lasers were gathered. Patients were evaluated and categorized according to their gender, sex, site of lesions, size of lesions, number of sessions, details of side effects, details of the VAS (Visual Analogue Scale) on third postoperative day, response of treatment to individual groups, time required for complete resolution, and details of recurrence. Inferential statistical analysis was performed.

**Results:**

We included 73 patients, of whom 43 and 30 received laser and sclerotherapy treatment, respectively. Compared with the sclerotherapy group, the laser group had less side effects including pain, edema, ulceration, ecchymosis, infections, and scarring. The difference in postoperative pain (VAS scale) between the groups was statistically significant (*p*-value 0.004). Complete remission was seen in the laser group, while 3 cases of the sclerotherapy group had no response (*p*-value −0.034). The laser group experienced greater recurrence than did the sclerotherapy group.

**Conclusions:**

Both sclerotherapy with laser and 3% sodium tetradecyl sulfate are effective for treating oral PG. Sclerotherapy is more effective in preventing recurrence. In terms of side effects, diode lasers are superior to sclerotherapy.

## 1. Introduction

Pyogenic granuloma (PG) is a tumor-like, non-neoplastic lesion of the soft tissue that commonly appears in the oral cavity. PG is not a granuloma but reactive inflammatory hyperplasia. This term itself is inaccurate because this entity does not contain any purulent material and histologically resembles a granuloma. [1] Various terminologies have been proposed, such as Crocker and Hartzell's disease, angiogranuloma, vascular epulis, pregnancy tumor, granuloma gravidarum, hemangiomatous granuloma, and granuloma telangiectacticum. Several studies have classified PG as a lobar capillary hemangioma from a histological standpoint [2, 3].

Patients with PG present with a raised or exophytic growth that is painless, smooth, lobulated, and frequently sessile or pedunculated. The ulcerated surface may be covered with a yellow fibrinous film and tends to bleed spontaneously or in response to little trauma. However, the surface can become fibrosed with time and thus not present with bleeding. Based on the growth's vascularity, the color ranges from red-pink to purple. The size ranges from few millimeters to centimeters. Although the gingiva is the most common location, the entity can be seen on the buccal mucosa, tongue, lips, palate, and retrocommissural region. [1, 3] Surgical excision together with removal of the causative irritant/source of trauma and, if necessary, oral prophylaxis is typically the chosen therapy. However, surgical excision can lead to bleeding, incomplete resection, recurrence, esthetic concerns, and functional impairment. Owing to these limitations, studies have proposed alternative methods, including cryosurgery, laser, electrodesiccation, curettage, sclerotherapy, and steroid injection [4, 5].

Laser has proven to be an effective therapy for oral soft tissue pathologies. It works on the principle of stimulated emission to emit light. [6] Carbon dioxide (CO_2_), pulsed dye, neodymium-doped yttrium aluminum garnet (Nd : YAG), diode, and erbium-doped yttrium aluminum garnet lasers (Er : YAG) have all been used to treat PGs. [7–11].

Sclerotherapy involves the injection of a sclerosant (tissue irritant). A sclerosant causes tissue irritation with endothelial damage, inflammation, and local tissue necrosis. Fibrosis and contracture eventually cause the lesion to disappear. [12] Because of their safety and efficacy, sodium tetradecyl sulfate (STS), monoethanolamine oleate, and polidocanol have been utilized as sclerosants in oral PG [13–15].

Although studies have used either sclerotherapy or laser for the treatment of oral PG, no study has compared between these techniques. This observational retrospective study compared the efficacy of laser and sclerotherapy for PG. The study results would contribute to the existing knowledge base.

## 2. Materials and Methods

This retrospective study was conducted in the Department of Oral Medicine and Radiology at Nobel Medical College Teaching Hospital, Biratnagar, Nepal. All study procedures were conducted as per World Medical Association's Declaration of Helsinki. The institutional ethics committee (Ref: IRC-NMCTH 569/2021) approved this study on January 10, 2021. The following study was performed as per the Strobe Statement (strobe-statement. org) [16].

### 2.1. Study Population, Setting, and Design

Patients who visited the Department of Oral Medicine and Radiology at Nobel Medical College Teaching Hospital, Biratnagar, Nepal, between July 2016 and January 2021 were included in this descriptive, retrospective, single-center study.

### 2.2. Inclusion Criteria

All patients clinically diagnosed as having PG received treatment with a diode laser and sclerotherapy with minimal 3-month follow-up were included.

### 2.3. Exclusion Criteria

Those with incomplete treatment and those lost to follow-up for at least 3 months were excluded.

### 2.4. Treatment Protocol

The clinical records of patients who received the same sclerotherapy and laser treatment protocol (as discussed below) were selected.

#### 2.4.1. Diode Laser Procedure (980 nm, IndiLase, India)

Perilesional local anesthetic infiltration was employed. A diode (Ga-Al-As, DILAS, Germany; model: IndiLase, MEDSOL, Hosur, India) operating in a continuous mode with a power of 2.0 W and a flexible 400-*μ*m diameter optic fiber (Polyamide, 400-*μ*m fiber made by MED-Fibers) fitted with a hand-piece were employed. The average amount of energy delivered to tissues was 1600 J/cm^2^. Aseptic conditions were maintained throughout, and both the patient and operator were asked to wear protective spectacles. In the contact mode, the laser fiber was used. To prevent recurrence, the remaining soft tissue close to the tooth was trimmed to ensure the complete lesion removal. Excisional samples were confirmed as PG through histological examination.

After laser surgery, postoperative instructions for wound care, diet, and postoperative drugs including nonsteroidal anti-inflammatory drugs were prescribed to all patients. To avoid subsequent infection, all patients received a topical antiseptic solution (betadine) for mouth rinse. Oral prophylaxis was administered to all patients after therapy completion. Patients were followed after 3 days, 1 week, 15 days, 30 days, and 3 months postoperatively.

#### 2.4.2. Procedure of Sclerotherapy with 3% STS

After the application of topical local anesthetic spray, undiluted 3% STS (60 mg/2 mL) was progressively injected with an insulin syringe into the base of the lesion. Multiple injections were used to treat larger lesions. The typical dosage was 0.1-0.3 mL of 3% STS. To avoid tissue necrosis and discomfort, PG of the palate was not treated with sclerotherapy.

Analgesics and anti-inflammatory drugs were prescribed to patients with inflammation. The patients were followed after 3 days, 1 week, 15 days, 30 days, and 3 months postoperatively. After treatment completion, oral prophylaxis was administered.

Depending on the desired outcome and extent of the lesion, laser treatment and sclerotherapy were performed in one to multiple sessions.

### 2.5. Confounders, Variables, and Assessment

Following variables were studied: age, sex, site of lesions, size of lesions, number of sessions, details of side effects, details of the VAS scale on the third postoperative day, the response of treatment to individual groups, the time required for complete resolution/healing, and details of recurrence.

### 2.6. Statistical Analysis

The collected data were evaluated using SPSS (version 24.0, IBM INC, Illinois, USA). The chi-squared test was employed for categorical data. A *P*-value of <0.05 was considered statistically significant.

## 3. Results

We identified 96 case records of oral PG treated with a diode laser or sclerotherapy. After applying the exclusion criteria, we recruited 73 cases; of these, 43 received treatment with a diode laser and 30 with sclerotherapy. Middle-aged women (21-45 years) were predominant in both laser and sclerotherapy groups. The patients' age ranged from 16 to 70 years (Table 1). The relationship between age (*P* = 0.572) and sex (*P*=0.809) was nonsignificant for the laser and sclerotherapy groups (Table 1).

No significant differences in sites and sizes of lesions were noted between the groups (*P*=0.844 and 0.401, respectively; Table 2).

Oral PG commonly involved the gingiva, followed by the lingual (tongue), lip, buccal mucosa, palate, labial mucosa, and retrocommissural areas (Table 2).

Most PG cases were treated with laser (81.3%) in a single session, but multiple sessions were required for sclerotherapy (83.4%) based on lesion size and response to treatment. More sessions in the sclerotherapy group could be because of the administration of few injections per session according to the routine protocol (Table 2).

**Figure 1:**
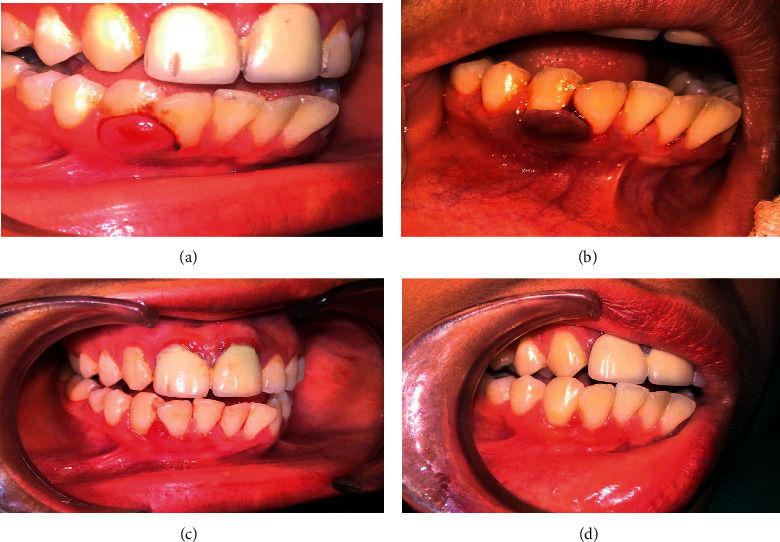
Case of pyogenic granuloma of gingival region treated with sclerotherapy with sodium tetradecyl sulfate (STS). (a) Presence of reddish pink sessile growth in relation with 42, 43. (b) Color changes soon after injection of 0.1 ml STS 3%. (c) 90% regression of the lesion. (d) Complete healing (proper scaling and root planning were advocated).

**Figure 2:**
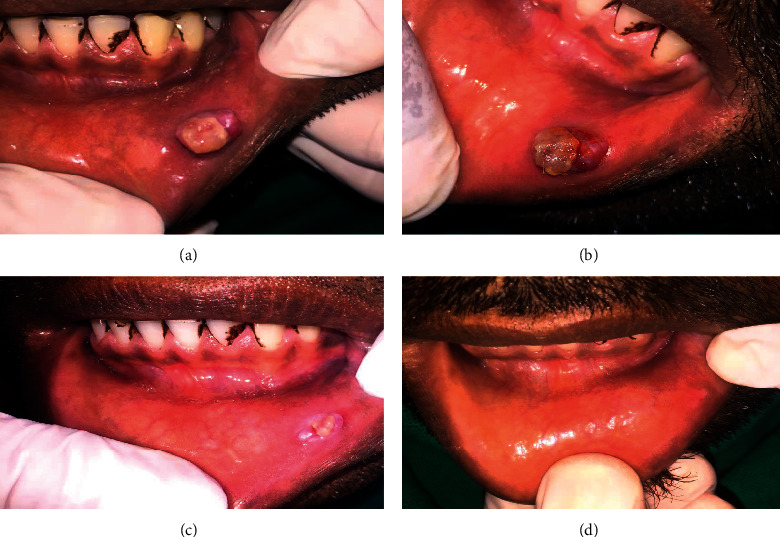
Another case of pyogenic granuloma of labial mucosa treated with sclerotherapy with sodium tetradecyl sulfate (STS). (a) Presence of reddish pink sessile growth on the tip of the tongue. (b) Intralesional color changes soon after injection of 0.2 ml STS 3%. (c) 90% regression of the lesion. (d) Complete healing.

**Figure 3:**
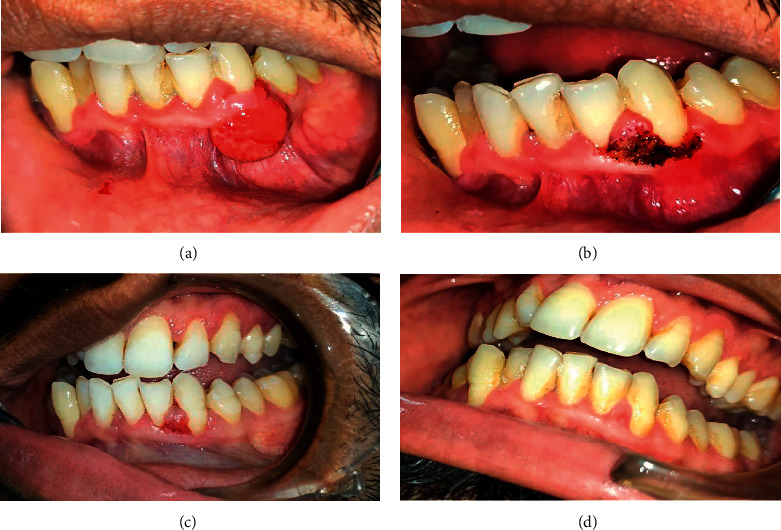
Pyogenic granuloma of gingival lesion treated with diode laser 980 nm (IndiLase). (a) Presence of reddish pink sessile growth in relation with 32 and 33. (b) Postoperative after laser excision. (c) Healing of the lesion. (d) Complete healing (proper scaling and root planning were advocated).

**Figure 4:**
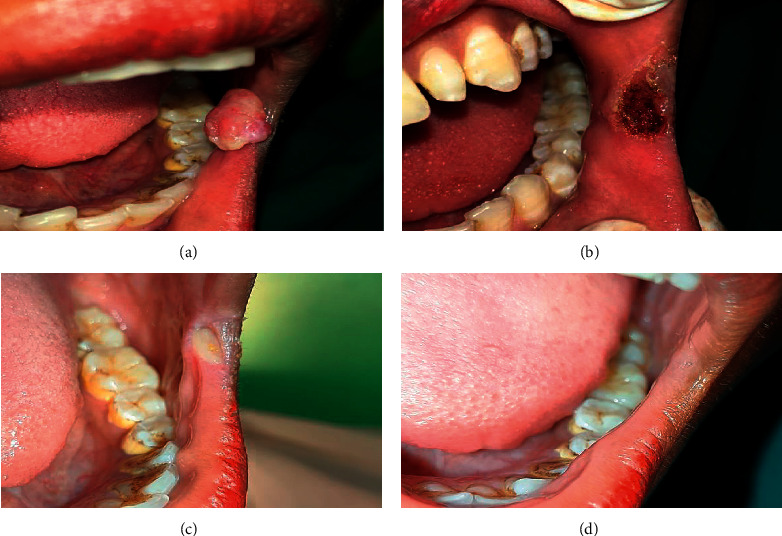
Another case of pyogenic granuloma of retrocommissural region treated with diode laser 980 nm (IndiLase). (a) Presence of reddish pink sessile growth. (b) Postoperative after laser excision. (c) 90% regression of the lesion. (d) Complete healing.

Compared with the sclerotherapy group, the laser group had few side effects. Little bleeding was noted in both groups. The difference in postoperative pain (VAS scale) between groups was significant (*P*=0.004). When we compared postoperative pain on third day postoperatively, 53.5% of patients in the laser group had mild pain (VAS = 1–3), whereas 73.3% in the sclerotherapy group had severe pain (VAS = 7–10). The difference was statistically highly significant (*P* < 0.001). Complete remission was seen in the laser group. Three cases in the sclerotherapy group exhibited no response (*P*=0.034). The diagnosis of these 3 cases was confirmed after the excision of lesions (Table 2; Figures 1–4).

The laser group had greater recurrence than did the sclerotherapy group, although the difference was nonsignificant. The majority of patients with PG of the lip had recurrence. Recurrence in the gingival area was noted in some patients. This might be attributable to either partial removal or failure to maintain proper dental hygiene (Table 2). Site and size of involvement, number of sessions, side effects, pain (VAS scale), response to treatment, and recurrence are summarized in Table 2.

## 4. Discussion

PG is a common, non-neoplastic reactive growth in the oral cavity. Low-grade local irritation, poor dental hygiene, traumatic damage, hormonal disturbances, and some medicines might trigger PG. [1] Vascular endothelial growth factor (VEGF), angiostatin, basic fibroblast growth factor (bFGF), and morphogenesis factors (angiopoietin-1, angiopoietin-2, tie-2, ephrinB2, and ephrinB4) were elevated in PG compared with the healthy gingiva [17, 18].

PG is known as a pregnancy tumor or granuloma gravidarum in pregnant women. Estrogen appears to enhance granulation tissue production by stimulating substances, such as nerve growth factor (NGF), basic fibroblast growth factor (bFGF), granulocyte macrophage-colony stimulating factor (GM CSF), transforming growth factor beta-1(TGF-*β*1), and VEGF (vascular endothelial growth factor) [1, 17, 18].

PG accounts for 26.8%-32% of all reactive lesions, with the peak incidence noted in the third decade; the patients' age ranges from 11 to 40 years. Women are more frequently affected with a predilection of 3 : 2 over men. [19] In this study, middle-aged women (21-45 years) were predominant in both the laser and sclerotherapy groups. [19].

Various treatment modalities have been discussed in the literature, including surgical excision, cryosurgery, curettage, electrodessication, chemical cautery, corticosteroid injection, sclerotherapy, and lasers. However, surgical excision has certain drawbacks; thus, necessitating the need for alternative methods [4, 5].

Laser therapy is among the most common treatments for oral PG. Pisano et al. indicated that laser is superior to surgical excision with advantages of absence of intraoperative and postoperative discomfort, pain, and scarring; less invasiveness; better postoperative management; effective hemostasis with better bleeding control; and improved patient compliance. [4] Other advantages of laser, such as minimum postoperative swelling, low mechanical trauma risk, and bactericidal properties, reduce the need for antibiotics postoperatively. Moreover, minimal local anesthesia for soft tissue treatments and minimal or no suture are required [6].

Less invasiveness, absence of intra- and postoperative discomfort and pain, effective hemostasis with better control of bleeding, absence of scarring, better postoperative management, and greater patient compliance are the advantages of laser therapy.

Disadvantages of laser therapy are (a) prolonged healing time owing to the sealing effect on blood vessels in the surgical field, (b) laser-induced ocular damage if preventive measures are not applied (e.g., the use of laser eyeglasses), (c) requirement of heavy pressure evacuation suction/smoke evacuator to avoid plume-induced health effects, and (e) expensive additional training [6].

Compared with other lasers, diode lasers are cheaper, compact in size, portable, and easier to handle in soft tissue surgeries. Moreover, they can be used in the contact mode [19].

Sclerotherapy has been used to treat various soft tissue lesions in the oral cavity, such as vascular lesions, lymphangioma, mucocele, and PG, due to its certain advantages such as noninvasiveness, low cost, safety, reliability, negligible blood loss, and no special requirement of postoperative dressings [5]. In PG, sclerotherapy may irreversibly injure endothelial cells comprising the major part of lesions and induce necrosis of the entire lesion, thereby eliminating recurrence risk [20].

The disadvantages of sclerotherapy include postoperative pain, edema, sloughing, ulceration, chances of ecchymosis, and tissue necrosis (Nicolau syndrome) [21]. Anaphylaxis can be a potential side effect of sclerotherapy; thus, patients should be inspected for possible allergic reactions. A patch test should be performed before the administration of a sclerosant [19]. We did not perform an allergic test but recorded any clinical sign of allergy if seen after the first injection. None of our patient had allergy to STS.

Compared with diode lasers, sclerotherapy results in more adverse reactions. Similar to a previous study [20], we noted severe postoperative pain, edema, ecchymosis, and tissue necrosis (Nicolau syndrome) in two patients (one in the gingival region and another in the lower lip region) in the sclerotherapy group. Edema was noted in patients with a lesion on the lip.

Both the techniques were significantly effective in oral PG treatment. Complete remission was noted in the laser group. Three patients in the sclerotherapy group had no response. This result is in contrast to that reported by Khaitan al [19] who reported complete PG regression after sclerotherapy with STS after 1-4 consecutive shots administered in a weekly interval. The unresponsive behavior of sclerosing agents might be due to lesion fibrosis [22].

Recurrence usually occurs due to incomplete removal or failure to maintain good oral hygiene. In our study, recurrence was not noted in any patient in the sclerotherapy group but in four (9.3%) patients in the laser group. Sclerosing agents act deeply and irreversibly compared with lasers. Shivhare [5], Khaitan, [19] and Samatha et al [23] have demonstrated the absence of recurrence after sclerotherapy.

Thus, our findings indicated the effectiveness of both techniques. Sclerotherapy was more effective with regard to side effects and improved patient compliance, whereas laser was better in terms of recurrence.

## 5. Limitations

Group allocation, treatment, and data collection were conducted by the same person; this could lead to bias. The sample size chosen was small, which could make the results inconclusive. A histopathological examination could not be performed for the sclerotherapy group owing to the study's retrospective nature. Comparing a surgical modality with a nonsurgical treatment modality for a common lesion could raise controversy.

## 6. Conclusion

We conclude that sclerotherapy with laser and sclerotherapy with 3% sodium tetradecyl sulfate are effective in the treatment of oral pyogenic granuloma. It offers an alternative to conventional methods like scalpel surgery. Diode laser seems to be better than sclerotherapy given fewer side effects and patient comfort; while for recurrent cases, sclerotherapy can be considered a better treatment choice. Further, studies on the comparison of different types of lasers can be conducted to determine effectiveness in treating pyogenic granuloma. Due to the scarcity of the literature on this topic, the authors recommend conducting similar studies on this topic to strengthen the evidence and provide guidelines for the choice of the best treatment modality for pyogenic granuloma [14].

## Figures and Tables

**Table 1:** Details of age and Gender.

	Laser group	STS group	Chi sq value	*P -*value
Details of age				
**12-20**	4 (9.3%)	1 (3.3%)	**1.092**	**0.572 NS**
**21-45**	27 (62.7%)	19 (63.4%)		
**>45**	12 (28%)	10 (33.3%)		
Total	43 (100%)	30 (100%)		

Details of gender				
Male	16 (37.2%)	12 (40%)	**0.058**	**0.809 NS**
Female	27 (62.8%)	18 (60%)		
Total	43 (100%)	30 (100%)		

**Table 2:** Details of comparison between diode laser and sclerotherapy.

	Laser group	STS group	Chi sq-	*P*-value
Details of site of involvement				
Gingiva	19(44.2%)	13 (43.3%)	**2.833**	**0.844 NS**
Tongue	8 (18.6%)	7 (23.3%)		
Buccal mucosa	3 (7%)	2 (6.7%)		
Upper lip	3 (7%)	3 (10%)		
Lower lip	2 (4.7%)	2 (6.7%)		
Lower labial mucosa	2 (4.7%)	1 (3.3%)		
Upper labial mucosa	1 (2.3%)	1 (3.3%)		
Palate	3 (7%)	0		
Retrocommissural area	2 (4.7%)	1 (3.3%)		
Total	43 (100%)	30 (100%)		

Details of size of involvement				
Less than 1.5 cm	22 (51.1%)	11 (36.6%)	**1.825**	**0.401 NS**
1.6-2.0 cm	18 (41.8%)	15 (50%)		
More than 2.0 cm	3 (7.1%)	4 (13.4)		
Total	43 (100%)	30 (100%)		

Details of the number of session				
1 session	35 (81.3%)	05 (16.6%)	**29.89**	**<0.001 ** ^*∗∗*^
More than 1 session	08 (18.6%)	25 (83.4%)		
Total	43 (100%)	30 (100%)		

Details of the side effects				
Bleeding	27 (62.8%) (minor bleeding)	17 (56.7%) (minor bleeding)	**0.277**	**0.599 NS**
Pain	33 (76.7%)	30 (100%)	**8.084**	**0.004 ** ^*∗*^
Edema	04 (9.3%)	10 (13.3%)	**6.584**	**0.010 ** ^*∗*^
Superficial ulceration	00	30 (100%)	**73**	**<0.001 ** ^*∗∗*^
Ecchymosis/necrosis	00	02(6.7%)	**2.947**	**0.086 NS**
Infection	00	02 (6.7%)	**2.947**	**0.086 NS**
Scarring	05 (11.6%)	02 (6.7%)	**7.694**	**0.006 ** ^*∗*^

Details of the VAS scale on the 3^rd^ day of treatment				
7-10 VAS	00	22 (7.37%)	**51.447**	**<0.001 ** ^*∗∗*^
4-6 VAS	15 (34.9%)	08 (26.7%)		
1-3 VAS	23 (53.5%)	00		
0 VAS/no pain	05 (11.6%)	00		
Total	43 (100%)	30 (100%)		

Response of treatment to individual groups				
Complete response	43 (100%)	27 (90%)	**4.484**	**0.034 ** ^*∗*^
Moderate response	00	00		
No response	00	03		
Total	43 (100%)	30 (100%)		

Time required for complete resolution/healing				
1 week	08 (18.6%)	00	**27.378**	**<0.001 ** ^*∗∗*^
2 weeks	23 (53.5%)	04 (13.3%)		
3 weeks	12 (27.9%)	21 (7.5)		
More than 3 weeks	00	05 (16.7%)		
Total	43 (100%)	30 (100%)		

Details of the recurrences based on site				
Recurrences	04 (9.3%)	00	**2.322**	**0.067 NS**
No recurrence	39 (90.7%)	30 (100%)		
Total	43 (100%)	30 (100%)		

## Data Availability

The data supporting the results of this study were obtained from the Department of Oral Medicine and Radiology, Nobel Medical College and Teaching Hospital, Biratnagar, Nepal. The data used are included within the article and may also be available by e-mail upon request to the corresponding author.

## Abbreviation

PG: Pyogenic granuloma
VAS: Visual Analogue Scale
CO_2_: Carbon dioxide
Nd: YAG: Neodymium-doped yttrium aluminum garnet
Er: YAG: Erbium-doped yttrium aluminum garnet lasers
STS: Sodium tetradecyl sulfate
VEGF: Vascular endothelial growth factor
bFGF: Basic fibroblast growth factor
NGF: Nerve growth factor
bFGF: Basic fibroblast growth factor
GM CSF: Granulocyte macrophage-colony stimulating factor
TGF-*β*1: Transforming growth factor beta-1
VEGF: Vascular endothelial growth factors.
